# Small-Molecule Acetylation Controls the Degradation of Benzoate and Photosynthesis in Rhodopseudomonas palustris

**DOI:** 10.1128/mBio.01895-18

**Published:** 2018-10-16

**Authors:** Chelsey M. VanDrisse, Jorge C. Escalante-Semerena

**Affiliations:** a Department of Microbiology, University of Georgia, Athens, Georgia, USA; Cornell University; University of Delaware; Max-Planck Institute, Marburg

**Keywords:** N-acetyltransferases, benzoate degradation, regulation of gene expression, small molecule acetylation

## Abstract

The degradation of lignin-derived aromatic compounds such as benzoate has been extensively studied in Rhodopseudomonas palustris, and the chemistry underpinning the conversion of benzoate to acetyl coenzyme A (acetyl-CoA) is well understood. Here we characterize the last unknown gene*, badL,* of the *bad* (benzoic acid degradation) cluster. BadL function is required for growth under photoheterotrophic conditions with benzoate as the organic carbon source (i.e., light plus anoxia). On the basis of bioinformatics and *in vivo* and *in vitro* data, we show that BadL, a Gcn5-related *N-* acetyltransferase (GNAT) (PF00583), acetylates aminobenzoates to yield acetamidobenzoates. The latter relieved repression of the *badDEFGAB* operon by binding to BadM, triggering the synthesis of enzymes that activate and dearomatize the benzene ring. We also show that acetamidobenzoates are required for the expression of genes encoding the photosynthetic reaction center light-harvesting complexes through a BadM-independent mechanism. The effect of acetamidobenzoates on pigment synthesis is new and different than their effect on the catabolism of benzoate.

## Abstract (precis)

This work shows that the BadL protein of Rhodopseudomonas palustris has *N-*acetyltransferase activity and that this activity is required for the catabolism of benzoate under photosynthetic conditions in this bacterium. R. palustris occupies lignin-rich habitats, making its benzoate-degrading capability critical for the recycling of this important, energy-rich biopolymer. This work identifies the product of the BadL enzyme as acetamidobenzoates, which were needed to derepress genes encoding benzoate-degrading enzymes and proteins of the photosynthetic apparatus responsible for the generation of the proton motive force under anoxia in the presence of light. In short, acetamidobenzoates potentially coordinate the use of benzoate as a source of reducing power and carbon with the generation of a light-driven proton motive force that fuels ATP synthesis, motility, transport, and many other processes in the metabolically versatile bacterium R. palustris.

## INTRODUCTION

**FIG 1:**
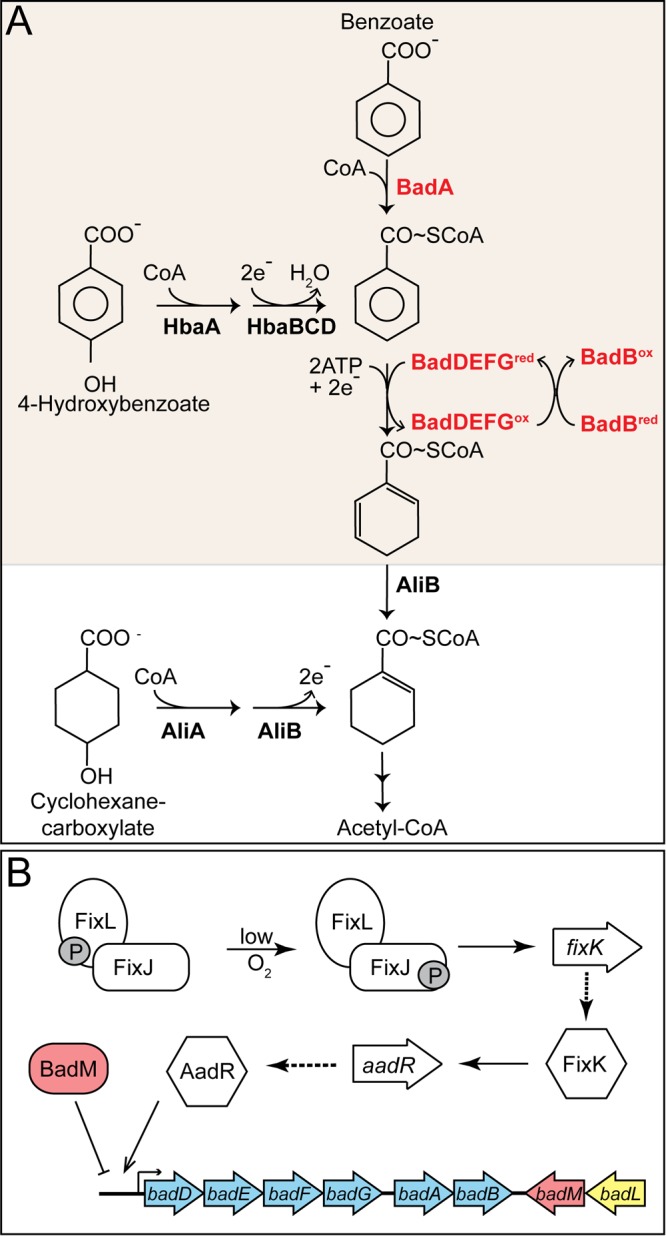
Aromatic catabolism and gene regulation in R. palustris. (A) Benzoate is activated to its CoA thioester by BadA. Hydroxybenzoate can also be converted to benzoyl-CoA in multiple steps by HbaA and HbaBC. The benzene ring of benzoyl-CoA is reduced by BadDEFG, yielding cyclohex-1,5-ene-carboxyl-CoA, which is further reduced by BadJ to cyclohex-1-ene-carboxyl-CoA. The latter is the entry point for cyclohexanecarboxylate into the pathway. Cyclohex-1-ene-carboxyl-CoA undergoes a ring reduction, ring cleavage, and several β-oxidations to eventually form acetyl-CoA. Arrows between cyclohex-1-ene-carboxyl-CoA and acetyl-CoA represent a simplified version of enzymatic reactions carried out by BadK, BadH, and BadI. BadA, benzoyl-CoA synthetase; HbaA, 4-hydroxybenzoyl-CoA synthetase; HcrABC, 4-hydroxybenzoyl-CoA reductase; BadDEFG, benzoyl-CoA reductase; BadB, ferredoxin; AliA, cyclohexancarboxyl-CoA synthetase; AliB, cyclohexancarboxyl-CoA dehydrogenase. Adapted from Harwood and Gibson (14). (B) Gene regulation of the *badDEFGAB* operon. Under low-oxygen conditions, phosphorylated FixL (FixL-P) relays its phosphate to FixJ, and FixJ-P activates expression of the global regulator, *fixK*. FixK activates *aadR* transcription, and AadR activates expression of the *badDEFGAB* operon (16). BadM is an Rrf2-like regulator that represses the *badDEFGAB* operon (15). The promoter region of BadM has been characterized and lies between *badD* and the upstream gene, *badC* (not shown). FixL, sensor histidine kinase; FixJ, response regulator; FixK, transcriptional regulator; AadR, Crp-like transcriptional activator; BadM, transcription factor.

Lignin is the second most abundant polymer in nature, second only to cellulose. Unlike cellulose, lignin does not contain carbohydrate monomers; instead, it is comprised of phenyl derivatives (e.g., coumaryl alcohol, syryngyl alcohol, and coniferyl alcohol). Lignin is found in the cell walls of plants, and it is estimated to represent approximately 25% of the terrestrial biomass (1). Aromatic compounds released from lignin are rich in energy and carbon; hence, it is not surprising that microbes that occupy environments rich in plant materials have evolved metabolic strategies for the degradation of such compounds. The purple nonsulfur photosynthetic alphaproteobacterium Rhodopseudomonas palustris is an aquatic bacterium that can degrade aromatic compounds into central metabolites. Shown in Fig. 1A is the pathway used by R. palustris for the degradation of aromatic compoundss under anoxic conditions (2). Other aromatic compounds such as chorismate, *p*-coumarate, toluene, vanillate, cresol, and phenol (to name a few; see reference 3 for a complete list of compounds) feed into the benzoate catabolism pathway via 4-hydroxybenzoate, benzoate, or benzoyl-coenyme A (CoA) (2, 3).

Benzoate catabolism has been studied in detail in R. palustris (3–11). The first step of the pathway is catalyzed by the benzoyl-CoA synthetase (BadA) (EC 6.2.1.25) enzyme, which activates benzoate to its CoA thioester, benzoyl-CoA (Fig. 1A) (7). The subsequent ring reduction of benzoyl-CoA to cyclohex-1,5-diene-1-carboxyl-CoA is catalyzed by the two [4Fe-4S]^+^, two [2Fe-2S]^+^ ATP-dependent reductase BadDEFG enzyme (EC 1.3.7.8) (Fig. 1A) (12, 13). The oxygen labile iron-sulfur centers of the reductase are rereduced by the ferredoxin protein BadB (1). Cyclohex-1,5-diene-1-carboxyl-CoA undergoes a series of reductions, ring cleavage, and β-oxidations, releasing acetyl-CoA and CO_2_ (2, 3, 14).

Due to the oxygen sensitivity and energetically demanding initial steps of benzoate catabolism (i.e., BadA, BadDEFG, and BadB), the genes encoding these proteins are tightly regulated by activators and repressors (4, 15). A majority of the genes required for benzoate catabolism are clustered within the genome of R. palustris (3). Specifically, the genes coding for the benzoyl-CoA synthetase (*badA*), benzoyl-CoA reductase (*badDEFG*), and ferredoxin (*badB*) comprise the *badDEFGAB* operon (Fig. 1B). The *badDEFGAB* operon is activated by AadR (a Crp-type family regulator) and repressed by BadM (a Rrf2-type regulator) (4, 15). The *aadR* gene is additionally regulated via activation by the oxygen-sensing two-component Fix (FixL, FixJ, and FixK) system (Fig. 1B) (16). The Fix-AadR hierarchy mediates the transition from microaerobic to anaerobic growth and further ensures anoxic conditions for the iron-sulfur center proteins of BadDEFG and BadB. To date, it is not known what signals lead to BadM derepression of *badDEFGAB.*

Within the aromatic degradation gene cluster, a single gene (*badL*) remains uncharacterized and is annotated as coding for a putative *N*-acetyltransferase. Acetylation occurs in all domains of life and involves the transfer of an acetyl group to the α or ε amino groups of proteins (*N*α or *N*ε) and amine groups (*N*α) of small molecules (17). Commonly, protein acetylation occurs on active site lysines of proteins (*N*ε), which in turn modulates their activity (18–25). Small-molecule acetylation has been shown to be involved in detoxification (26), translation inhibition (27, 28), and antibiotic neutralization (29, 30).

This study identifies BadL as a small-molecule *N-*acetyltransferase that modifies aminobenzoates (ABAs). We show that acetylated aminobenzoates (ABA^Ac^, also known as acetamidobenzoate) bind to BadM and that BadM/ABA^Ac^ complexes no longer repress *badDEFGAB* operon expression. Results of growth analyses of wild-type and mutant strains, quantitative reverse transcription-PCR (qRT-PCR), and electrophoretic mobility shift assays suggest that acetamidobenzoates play a role in the regulation of benzoate degradation in R. palustris. High-performance liquid chromatography (HPLC) coupled to tandem mass spectrometry (LC/MS/MS) data show that BadL acetylates ABAs, and electrophoretic mobility shift assays (EMSAs) show that BadM can bind all three forms of acetamidobenzoates. On the basis of our data, we conclude that BadL activity is required for R. palustris growth on benzoate, and we suggest that BadL may work as a sensing mechanism for the presence of ABAs in the environment. Surprisingly, acetamidobenzoates also affect the synthesis of light-harvesting complexes I and II of R. palustris. These results suggest that BadL may be a link between carbon utilization and the generation of a light-driven proton motive force. To our knowledge, this may be the first example of a Gcn5-type acetyltransferase connecting carbon and energy conservation in a photosynthetic bacterium.

## RESULTS

### BadL acetyltransferase activity is required for photoheterotrophic growth on benzoate.

**FIG 2:**
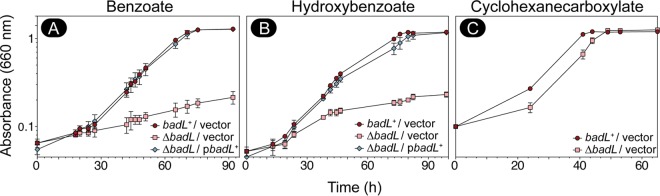
The BadL acetyltransferase is required for photoheterotrophic growth on benzoate and hydroxybenzoate. Cells were grown in biological triplicate photoheterotrophically on the carbon source (3 mM) listed above each panel, and growth was monitored at OD_660_. Cells lacking BadL had lower growth rates compared to *badL*^+^ controls when grown with benzoate (A) (pink squares versus red circles) or hydroxybenzoate (B) (pink squares versus red circles), but not when grown with cyclohexancarboxylate (C). Growth rates were restored when *badL* was reintroduced on a plasmid (blue diamonds). Each experiment was repeated in triplicate, and a representative growth curve is shown.

Prior to this work, the function of the putative BadL protein was unknown. Given the genomic context of the *badL* gene, we began investigating the function of this protein by deleting *badL* and screening for phenotypes related to benzoate utilization. The growth behavior of the R. palustris *badL* strain was assessed under photoheterotrophic conditions. An R. palustris Δ*badL* strain failed to grow photosynthetically on benzoate (Fig. 2A) or 4-hydroxybenzoate (Fig. 2B) compared to *badL*^+^ controls. Notably, the Δ*badL* strain grew as well as the *badL^+^* strain on cyclohexanecarboxylate (Fig. 2C), suggesting that BadL function was necessary only for substrate activation and ring reduction (Fig. 1A, shaded area). The enzymes catalyzing the early steps of the pathway include the benzoyl-CoA synthetase (BadA) (EC 6.2.1.25), a benzoyl-CoA reductase (BadDEFG [dearomatizing]) (EC 1.3.7.8), and ferredoxin (BadB). The above-mentioned genes comprise the *badDEFGAB* operon of R. palustris and are tightly regulated by activation and repression (Fig. 1B). On the basis of the results shown in Fig. 2, we hypothesized that BadL played an as-yet-unidentified role in the regulation of *badDEFGAB* expression and that its putative function was to acylate either a protein or a small molecule.

### Deletion of *badM* restores photoheterotrophic growth of a Δ*badL* strain on benzoate.

**FIG 3:**
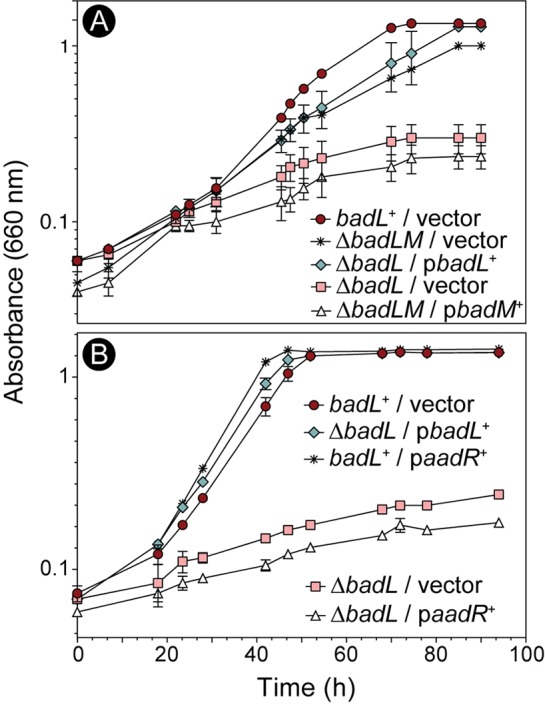
BadL-dependent phenotypes are restored upon deletion of *badM*, but not overexpression of *aadR*. Cells were grown in biological triplicate photoheterotrophically on benzoate (3 mM), and growth was monitored at OD_660_. (A) A Δ*badM* Δ*badL* strain grew similar to the *badL*^+^ strain (red circles versus asterisks), and ectopic expression of *badM^+^* in the Δ*badM* Δ*badL* strain restored repression, hence a lack of growth (triangles). (B) Overexpression of *aadR* in a Δ*badL* strain did not recover growth (white triangles). Each experiment was performed in triplicate, and a representative growth curve is shown. 4-ABA was present in the growth medium at 11 µM (2 mg/liter).

To address the possible involvement of BadL in *badDEFGAB* expression, we deleted *badM* in a Δ*badL* strain. In the absence of BadL, growth of R. palustris was restored when *badM* was deleted (Fig. 3A, asterisks). A Δ*badL* Δ*badM* strain carrying a plasmid encoding the wild-type allele of *badM* failed to grow photoheterotrophically on benzoate (Fig. 3A, triangles), suggesting that somehow BadL function affected BadM DNA-binding activity.

Importantly, overexpression of *aadR*, the activator of *badDEFGAB*, did not restore growth of the Δ*badL* strain on benzoate (Fig. 3B, triangles), leading us to hypothesize that either BadL directly acetylated BadM, or alternatively, BadL acetylated a small-molecule effector of BadM. We note that prior to this work, small-molecule effectors of BadM DNA-binding activity were not known (15).

**FIG 4:**
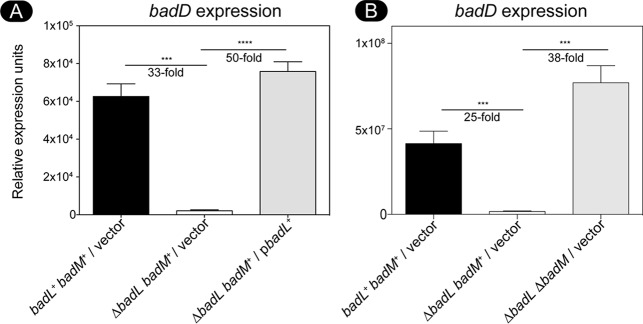
In a *badL* strain, *badDEFGAB* operon expression is reduced. Total RNA was obtained from cells grown photosynthetically with succinate and benzoate. (A) Expression of the *badDEFGAB* operon was assessed in *badL^+^* and Δ*badL* strains using qRT-PCR. The absence of *badL* led to a 30-fold decrease in *badD* expression. When *badL* was reintroduced on a plasmid, transcription increased 50-fold. (B) Expression of *badDEFGAB* was compared in *badM*^+^ or Δ*badM* strains. Cells lacking *badL* had 25-fold-lower transcription of *badDEFGAB*. If *badM* was also deleted in cells lacking *badL*, *badDEFGAB* transcription was restored. Transcripts of *badD* were normalized to transcripts of the housekeeping gene *fixJ*. Cells were grown in biological triplicate, and qRT-PCR was performed in technical triplicates of each biological replicate. Error bars represent the standard deviations of the means. Values that are significantly different are indicated by a bar and asterisks as follows: ***, *P* value of >0.0005; ****, *P* value of <0.0001.

We investigated the potential role of BadL in *badDEFGAB* expression. For this purpose, we used qRT-PCR to monitor *badDEFGAB* operon expression using primers and conditions described elsewhere (15). When R. palustris was grown photoheterotrophically on succinate supplemented with benzoate, cells lacking *badL* had 30-fold-less *badDEFGAB* transcript than *badL*^+^ strains (Fig. 4A). Additionally, deletion of *badM* in a Δ*badL* strain restored transcription of *badDEFGAB* under photoheterotrophic conditions with succinate plus benzoate (Fig. 4B). These results supported the idea that BadL was involved in the regulation of *badDEFGAB* expression, but it was unclear how.

### BadL homologues acetylate aminobenzoates, and the resulting acetamidobenzoates bind to BadM derepressing *badDEFGAB* expression.

**FIG 5:**
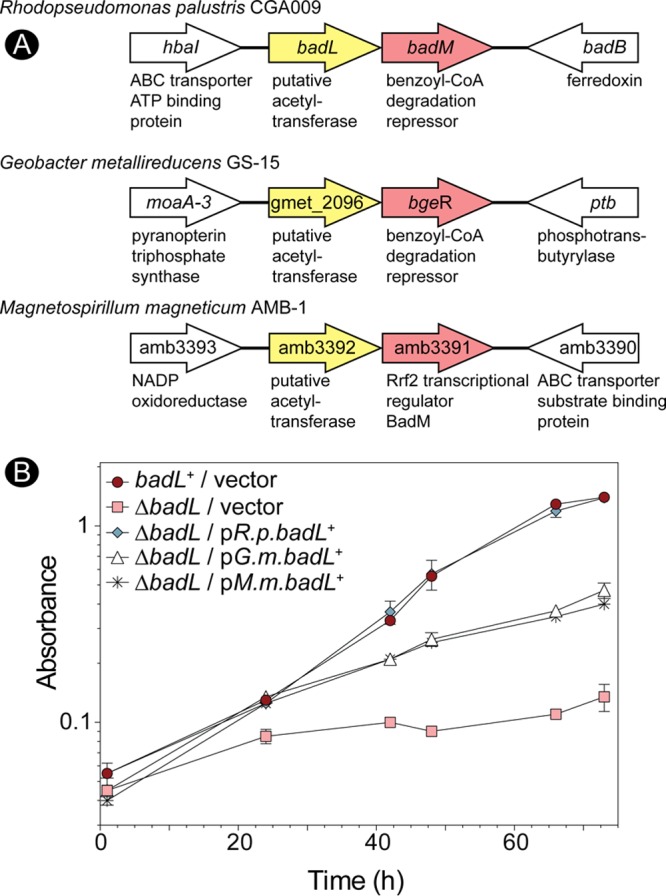
Bioinformatic analysis reveals putative *badL badM* gene clustering in other proteobacteria. (A) By analyzing the R. palustris *badL* and *badM* features of The SEED Viewer (pubseed.theseed.org/), other organisms were found to have similar clustering of Rrf2-type transcriptional regulators in putative operons with putative acetyltransferases. The putative BadL acetyltransferases from *Geobacter metallireducens* and *Magnetospirillum magneticum* were used for subsequent *in vitro* experiments. Genes surrounding the upstream and downstream *badL badM* regions for each organism are shown, and genes are not drawn to scale. (B) Cells were grown in biological triplicates photoheterotrophically with benzoate (3 mM). Cells lacking BadL complemented with *G. metallireducens badL* (*G.m.badL*) or *M. magneticum badL* (*M.m.badL*) had lower cell densities than *badL*^+^ controls did and recovered growth only partially.

Attempts to isolate reliably active R. palustris BadL (*Rp*BadL) protein were unsuccessful. While *Rp*BadL acetylated the small-molecule substrates mentioned below in subsequent experiments, the activity was inconsistent. For this reason, we used SEED viewer version 2.0 (31) to identify BadL homologues that clustered with BadM repressors. We focused our attention on BadL homologues present in *Magnetospirillum magneticum* (*Mm*BadL; locus tag amb3392, 41% identity to *Rp*BadL) and Geobacter metallireducens (*Gm*BadL; locus tag gmet_2096, 47% identity to *Rp*BadL) (Fig. 5A) because they shared the same genomic context and shared the highest level of identity. Both of these bacteria have been shown and predicted to degrade benzoate (32–36). Additionally, the genes coding for *Mm*BadL or *Gm*BadL partially complemented an R. palustris Δ*badL* strain, indicating they may have similar functions in *M. magneticum* and *G. metallireducens*, respectively (Fig. 5B)*. Mm*BadL and *Gm*BadL were successfully isolated and used in subsequent *in vitro* analyses. Attempts to acetylate BadM with *Mm*BadL or *Gm*BadL did not yield BadM^Ac^ under the conditions tested (data not shown).

**FIG 6:**
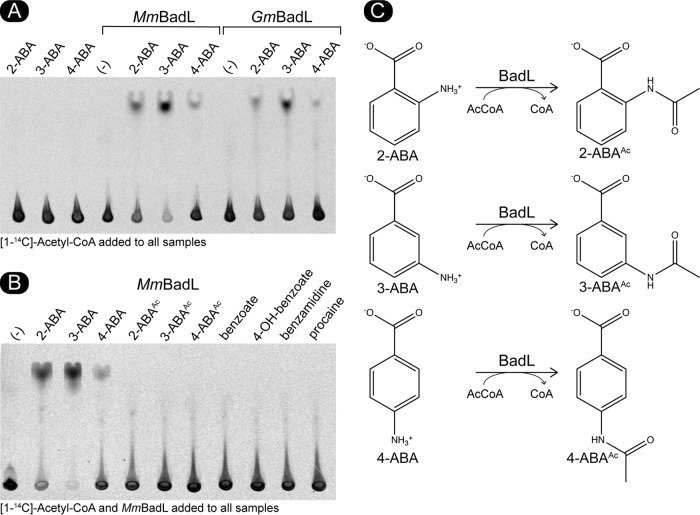
BadL homologues acetylate aminobenzoates *in vitro*. (A) *Mm*BadL and *Gm*BadL (3 μg) were incubated with [1-^14^C]acetyl-CoA and 2-aminobenzoate (2-ABA), 3-aminobenzoate (3-ABA), or 4-aminobenzoate (4-ABA). As a negative control, 2-ABA, 3-ABA, and 4-ABA were incubated with [1-^14^C]Ac-CoA (three leftmost lanes). The lanes designated by (-) indicate reaction mixtures that contained BadL and [1-^14^C]Ac-CoA but lacked substrate. The reaction mixtures were spotted onto the gels, and acetylated products migrated away from the origin where [1-^14^C]-Ac-CoA remained. (B) *Mm*BadL was incubated with various aromatic substrates as listed above each lane. Lanes labeled as 2-ABA^Ac^, 3-ABA^Ac^, and 4-ABA^Ac^ indicate reaction mixtures containing authentic acetylated 2-ABA, 3-ABA, and 4-ABA as described in Materials and Methods. Lane designated by (-) identify a reaction mixture where BadL was incubated with [^14^C-1]Ac-CoA but no substrate. The reaction mixtures were spotted onto gels, and acetylated products migrated away from the origin, where [1-^14^C]Ac-CoA remained. Images were acquired by exposure to a phosphor screen and subsequent imaging. (C) Proposed reaction schematic of BadL-mediated aminobenzoate acetylation.

We considered the possibility that the substrate of BadL was not a protein but a small molecule. For this purpose, we incubated putative small-molecule substrates structurally related to benzoate. The transfer of radiolabeled acetyl moieties was assessed by thin-layer chromatography (TLC) using a mobile phase that resolved substrates and acetyl-CoA from products. Radiolabel distribution on the TLC was visualized by phosphor imaging. As mentioned above, we tested benzoate derivatives that could be found in the environment occupied by this bacterium. One such molecule, 4-aminobenzoate, is routinely added to the growth medium (11 µM) as a precursor of folate synthesis, because the genome of R. palustris does not code for enzymes to synthesize its own 4-aminobenzoate. Using this approach, we found that *Mm*BadL and *Gm*BadL acetylated 4-aminobenzoate producing 4-acetamidobenzoate (Fig. 6A). We further tested whether BadL could also acetylate 2- and 3-aminobenzoate. In fact, *Mm*BadL and *Gm*BadL did acetylate 2-, 3-, and 4-aminobenzoate, yielding 2-, 3-, and 4-acetamidobenzoate, albeit to different extents. These results suggested that BadL acetylated the amino group of aminobenzoates. In contrast, *Mm*BadL and *Gm*BadL did not acetylate benzoate, benzoates with hydroxyl group substituents at the same positions, or acetamidobenzoates (Fig. 6B). The proposed reactions and their products are shown in Fig. 6C. When analyzing unreacted [1-^14^C]-acetyl-CoA ([1-^14^C]Ac-CoA) at the point of sample application on the TLC plate, it appeared as if *Mm*BadL had a preference for 3-ABA *in vitro* (Fig. 6A). We attempted to establish substrate preference by kinetic means, but unfortunately, the levels of activity of *Mm*BadL and *Gm*BadL were lower than the background limits of our assay; thus, we could not evaluate kinetic *Mm*BadL and *Gm*BadL parameters to determine a preferred substrate.

### Mass spectrometry analysis of the product of the *Mm*BadL reaction.

**FIG 7:**
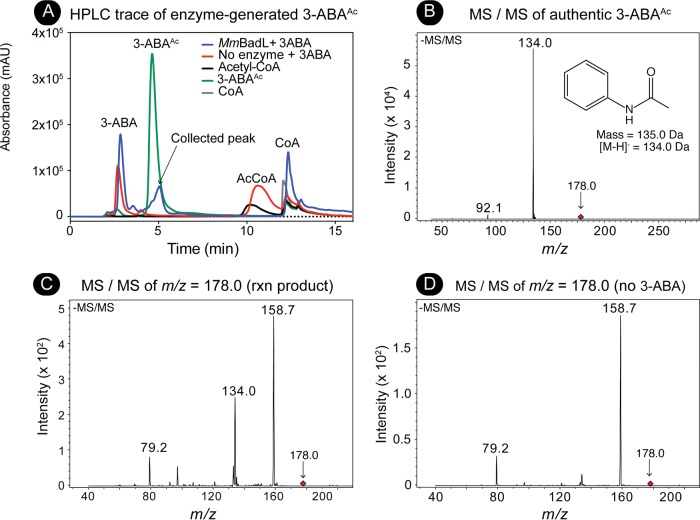
Isolation and identification of the *Mm*BadL reaction product by HPLC and mass spectrometry. (A) Reaction mixtures containing *Mm*BadL, 3-ABA, and Ac-CoA were set up as described in Materials and Methods. Protein was removed from mixtures, and material elution from a C_18_ kinetex column was monitored at 254 nm. Reaction mixtures containing *Mm*BadL were compared to known standards (3-acetamidobenzoate [green peak]), and the black arrow indicated fractions that were collected for MS/MS. Absorbance is shown in milli arbitrary units (mAU) on the y axis. (B) Mass spectrometry was performed with commercially available 3-acetamidobenzoate (1 mM), which was identified by the signal with an *m/z* of 178.0 Da (red diamond) in the negative ion spectrum shown. The 178.0-Da compound was fragmented (negative ion shown). The inset shows the structure of the compound with a mass of 134.0 Da, which was consistent with 3-acetamidobenzoate without the carboxylic acid. (C) Mass spectrometry was performed on the fraction collected in panel A. The compound with an *m/z of* 178.0 Da (red diamond) corresponding to 3-acetomidobenzoate was fragmented (negative ion spectrum shown). The peak at 134.0 Da corresponds to the structure in the inset in panel B. Signals observed at *m/z* values of 158.7 Da and 79.2 Da were determined to be background from machine, as indicated in panel D. rxn product, reaction product.

Since it appeared that *Mm*BadL acetylated 3-ABA more efficiently and 3-ABA^Ac^ (retention time = 5.0 min) was readily resolved by reverse-phase HPLC away from 3-ABA (retention time = 2.8 min) (Fig. 7A), the products of reaction mixtures containing *Mm*BadL and 3-ABA were analyzed by mass spectrometry (MS and MS/MS). A signal corresponding to the mass of enzyme-generated 3-ABA^Ac^ (*m/z = *178.0 Da) (see Fig. S1 in the supplemental material) was fragmented (Fig. 7C) and compared to the authentic standard (Fig. 7B). *Mm*BadL-generated and commercially available 3-ABA^Ac^ retained the same fragmentation pattern (*m/z *=* *134.0), a fragment that corresponded to the loss of the carboxylate moiety (Fig. 7B, inset). These results confirmed the identity of the *Mm*BadL product as 3-ABA^Ac^. A control experiment (solvent without HPLC product) was performed, and the signals with *m/z* values of 79.2 and 158.7 in Fig. 7B were found to be unrelated to the product of the reaction (Fig. 7D).

10.1128/mBio.01895-18.2 FIG S1 Mass spectrometry of 3-ABA^Ac^, 3-ABA, and BadL reaction product. Mass spectrometry (electrospray ionization [ESI]) was performed with commercially available 3-acetamidobenzoic acid or 3-aminobenzoic acid (1 mM), which was identified by the signal with an *m/z* of 178.0 Da or 137.1 Da, respectively. The inset shows the structure of each compound. MS/MS of BadL acetylated 3-ABA (as shown in Fig. 7) was performed on HPLC- purified product (right panel). Download FIG S1, TIF file, 9.5 MB. Copyright © 2018 VanDrisse and Escalante-Semerena.. 2018 VanDrisse and Escalante-Semerena This content is distributed under the terms of the Creative Commons Attribution 4.0 International license.

### Acetamidobenzoates bind to BadM lowering its affinity for the *badDEFGAB* promoter.

**FIG 8:**
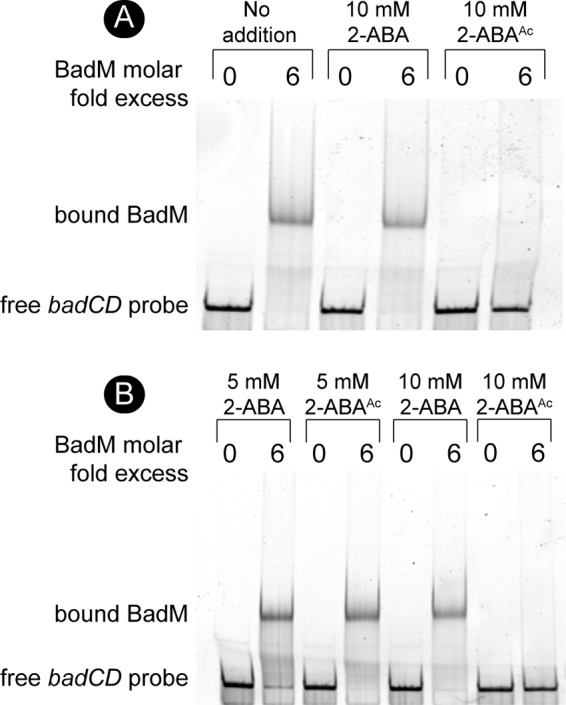
BadM DNA binding to the *badDEFG* promoter is reduced in the presence of acetylated 2-aminobenzoate. (A) Binding of BadM (72 nM) to the *badDEFGAB* promoter (12 nM) was analyzed by electrophoretic mobility shift assays using 6-FAM 5′-labeled probes. (A) Probe containing the intergenic region of *badCD* (212 bp, 0.303 pmol) was incubated with BadM (1.8 pmol) or without BadM. The addition of 2-aminobenzoate or 2-acetamidobenzoate is indicated above the respective lanes. (B) Binding of BadM to the *badCD* intergenic region as in panel A, but with different concentrations of 2-aminobenzoate or 2-acetamidobenzoate.

**FIG 9:**
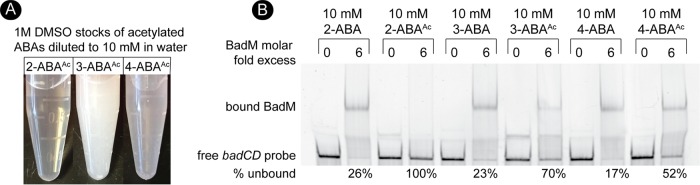
Binding of BadM to DNA is affected by all acetamidobenzoates. (A) To meet reaction volumes, 2-, 3-, and 4-acetamidobenzoate were resuspended in 100% DMSO to a final concentration of 1 M. The images show the solubility of each chemical when diluted to 10 mM in water. (B) 6-FAM 5′-labeled probe containing the intergenic region of *badCD* (0.303 pmol, 12 nM) was incubated with BadM (1.8 pmol, 72 nM) or without BadM. Various acetamidobenzoates were added to reaction mixtures to the final concentrations indicated above each lane. The percentage of unbound *badCD* probe was analyzed by comparing the intensities of upper and lower bands of samples containing BadM. Intensities were calculated using ImageQuant software, and the percentages correspond to the lanes directly above the values.

The binding of BadM to the *badDEFGAB* promoter has been analyzed by others (15). We synthesized the *badDEFGAB* promoter region (212 nucleotides, −191 to +21 of *badD* ATG) with a 6-carboxyfluorescein (6-FAM) fluorescent label covalently attached to the 3′ end of the probe. Under the conditions tested, BadM bound all of the fluorescently labeled probes at sixfold molar excess, and this condition was used to test the effects of 2-, 3-, and 4-acetamidobenzoate. When BadM was incubated with 2-ABA, its binding to the *badDEFGAB* promoter probe did not differ from that of BadM incubated with the probe in the absence of ABAs (Fig. 8A, compare lane 2 to lane 4). However, addition of 2-acetamidobenzoate blocked binding of BadM to its DNA target (Fig. 8A, compare lane 2 to lane 6). This change was clear when the concentration of 2-acetamidobenzoate in the reaction mixture was 10 mM; when the concentration of 2-acetamidobenzoate was 5 mM, BadM/*badDEFGAB* promoter probe interactions were unaffected (Fig. 8B). This information helped us assess the effects of 3- and 4-acetamidobenzoate on BadM/*badDEFGAB* promoter probe interactions. Notably, at the concentrations necessary to affect BadM/*badDEFGAB* promoter probe interactions, the solubility of 3- and 4-acetoamidobenzoate was substantially reduced relative to that of 2-acetoamidobenzoate (Fig. 9A). When incubated under the same conditions, BadM/*badDEFGAB* promoter probe interactions changed upon incubation with 3- and 4-acetoamidobenzoate, albeit to a lesser degree than with 2-acetoamidobenzoate (Fig. 9B, see free probe band percentages). The alluded solubility issues with 3-acetoamidobenzoate and 4-acetoamidobenzoate prevented the quantification of ligand effects of BadM’s DNA-binding activity. It is possible that all three acetamidobenzoates may serve as ligands for BadM. In combination with qRT-PCR data shown in Fig. 4, these results indicate that BadL acetylates aminobenzoates, which in turn bind to BadM, leading to derepression of the *badDEFGAB* operon.

### Addition of acetamidobenzoates to benzoate medium restores growth of cells devoid of BadL.

**FIG 10:**
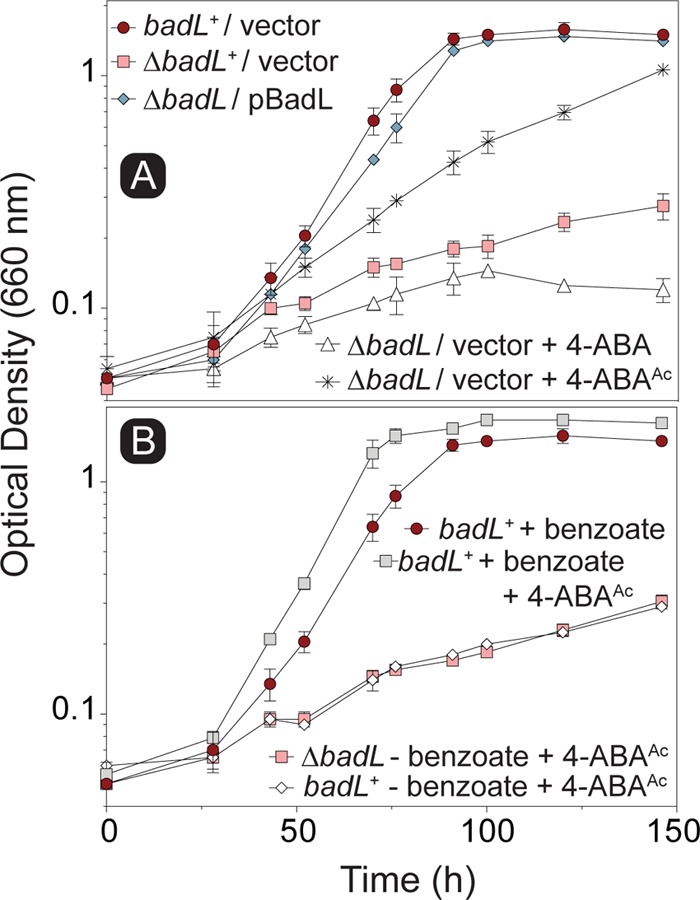
Addition of 4-acetamidobenzoate restores photoheterotrophic growth of Δ*badL* cells on benzoate. Cells were grown in biological duplicates photoheterotrophically on benzoate (3 mM) with or without acetamidobenzoates (5 mM), and growth was monitored at 660 nm. (A) Growth analysis of Δ*badL* cells was assessed upon the addition of 4-acetamidobenzoate. (B) To determine whether growth restoration of Δ*badL* cells was due to alternative carbon source utilization, *badL^+^* and Δ*badL* cells were grown on 4-acetamidobenzoate in the presence or absence of benzoate, as indicated in the symbol key within each panel. Each experiment was performed in triplicate, and a representative growth curve is shown. Error bars represent standard deviations (SD) of a biological triplicate. 4-ABA was present in the growth medium at 11 µM (2 mg/liter).

To assess the substrate specificity of BadM *in vivo*, authentic 2-, 3-, or 4-acetamidobenzoate was added to cultures of a Δ*badL* strain, and photoheterotropic growth with benzoate as the carbon source was compared to the response of the same strain to nonacetylated aminobenzoate. When 4-acetamidobenzoate was added to the medium, the final density of the Δ*badL* cultures was similar to that of the *badL*^+^ strain, albeit growth rates were different (Fig. 10A). We note that restoration of growth of the Δ*badL* strain upon addition of 4-acetamidobenzoate was not due to alternative carbon source utilization, as *badL*^+^ and Δ*badL* cells failed to grow when only 4-acetamidobenzoate was added to the medium (Fig. 10B). Notably, the effect of 2- or 3-acetamidobenzoate on the growth of the Δ*badL* strain was erratic and was not studied further (data not shown). Of note, the addition of 4-acetamidobenzoate decreased the lag phase of *badL*^+^ cells by 20% to 40% but did not alter the doubling time of these cells (from Fig. 10).

### BadL plays a role in the expression of genes encoding reaction center proteins.

**FIG 11:**
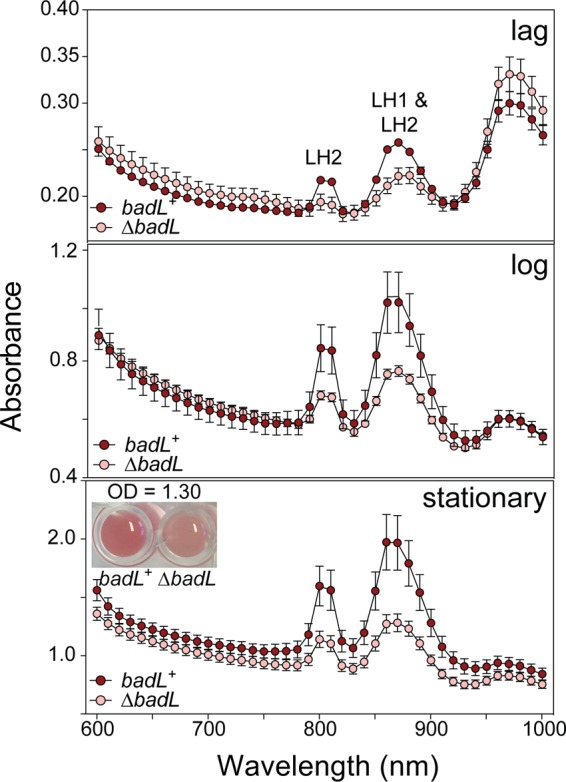
Cells lacking BadL have reduced light-harvesting reaction center complex pigments. The amount of light-harvesting 1 (LH1) and light-harvesting 2 (LH2) reaction center complexes in *badL*^+^ and Δ*badL* strains was monitored spectrophotometrically during lag, log, and stationary growth phases. LH1 complexes absorb at 880 nm, and LH2 complexes absorb at 808 and 863 nm. Cells were analyzed at similar cell densities (measured at 660 nm) to ensure that differences in complexes were not due to differences in cell number. Data were obtained in triplicate on a SpectraMax instrument with absorbance readings every 10 nm. For visualization purposes, the inset in the bottom panel shows *badL*^+^ and Δ*badL* cells at identical stationary-phase OD_660_. The experiment was repeated in biological triplicates, and error bars represent SD of a technical triplicate. 4-ABA was present in the growth medium at 11 µM (2 mg/liter).

We noticed a substantial difference in pigmentation between *badL*^+^ and Δ*badL* strains during photoheterotrophic growth with benzoate. To determine whether this observation was due to differences in cell density or pigment synthesis, R. palustris *badL*^+^ and Δ*badL* strains were grown on benzoate, and peak intensities at wavelengths corresponding to the light-harvesting 1 (LH1) reaction center complex (880 nm) and the light-harvesting 2 (LH2) reaction center complex (808 nm and 863 nm) were analyzed. Absorbance scans (*A*_600_ to *A*_1000_ [*A*_600–1000_]) of cultures of *badL*^+^ and Δ*badL* strains were obtained during lag, log, and stationary growth phases. The R. palustris Δ*badL* strain grew at a much lower rate with benzoate compared to the *badL^+^* strain (9-h versus 36-h doubling time [Fig. 2]), and it was therefore ensured that comparisons between *badL*^+^ and Δ*badL* were presented at the same cell densities. When the pigments of LH1 and LH2 complexes were analyzed, we found that Δ*badL* cells had less pigment in both complexes than *badL*^+^ cells (Fig. 11). The lower level of pigment in the Δ*badL* strain was independent of growth phase and was seen in lag, log, and stationary growth phase (Fig. 11). This lighter pigmentation was most evident in stationary-phase cells, as shown in the inset of Fig. 11.

**FIG 12:**
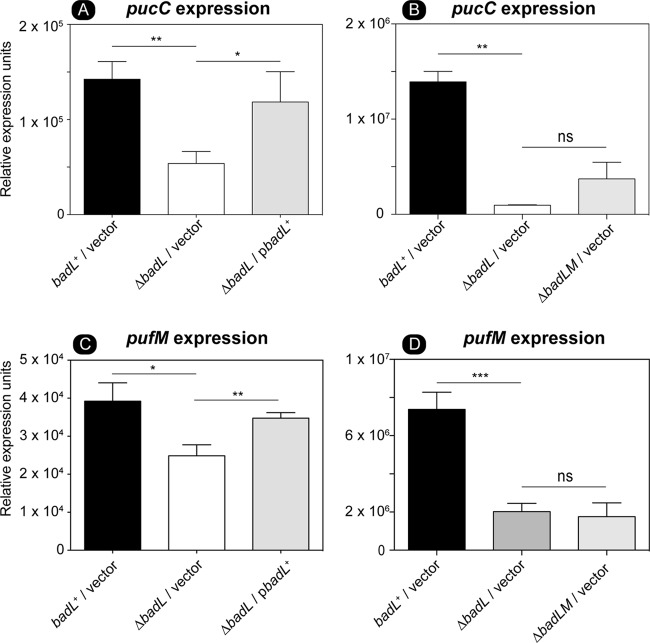
Deletion of *badL* lowers the level of *pucC* RNA transcripts, an effect that is independent of the presence or absence of BadM. Total RNA of cells grown photosynthetically with succinate plus benzoate was harvested as described in Materials and Methods. Expression of *pucC* was assessed in *badL^+^*, Δ*badL,* or Δ*badL* Δ*badM* strains using qRT-PCR. (A) The absence of *badL* led to a decrease in *pucC* transcription. The gene for *pucC* codes for a probable major facilitator superfamily transporter for photosynthetic complex assembly. Transcription of *pucC* is restored when *badL* was provided on a plasmid. (B) *pucC* RNA level was not significantly different in Δ*badL* and Δ*badL* Δ*badM* strains. (C) Deletion of *badL* led to a decrease in *pufM* transcription. The gene for *pufM* codes for the photosynthetic reaction center complex M. Transcription of *pufM* is restored with *badL* on a plasmid. (D) *pufM* RNA level was not significantly different in a Δ*badL* versus Δ*badL* Δ*badM* strain. Values that are significantly different are indicated by a bar and asterisks as follows: **, *P* value of >0.005; *, *P* value of <0.01. Values that are not significantly different are indicated by a bar labeled ns.

**FIG 13:**
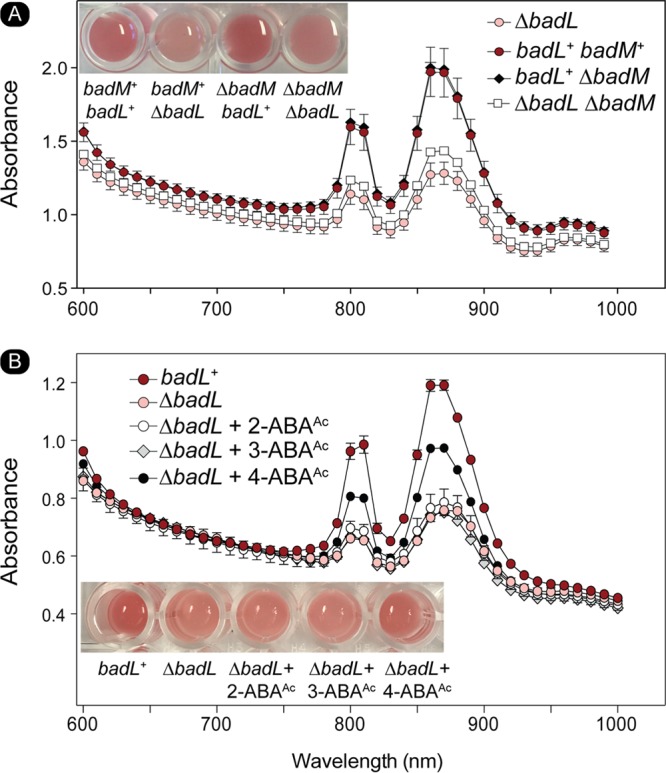
The absence of BadM does not restore pigment synthesis in a Δ*badL* strain. The content of light-harvesting 1 (LH1) and light-harvesting 2 (LH2) reaction center complexes was assessed spectrophotometrically. LH1 complexes absorb at 880 nm, and LH2 complexes absorb at 808 and 863 nm. Cells were analyzed at similar cell densities (measured at OD_660_) to ensure that differences in complexes were not due to differences in cell numbers. Data were obtained in triplicate on a SpectraMax spectrophotometer with absorbance readings every 10 nm. (A) LH1 and LH2 complex analysis for *badL*^+^ and Δ*badL,* Δ*badM,* and Δ*badL* Δ*badM* cells. Picture inset shows the coloration of cells in a microtiter dish. (B) LH1 and LH2 complex analysis for *badL*^+^ and Δ*badL* cells with either 2-, 3-, or 4-acetamidobenzoate (5 mM). 4-ABA was present in the growth medium at 11 µM (2 mg/liter).

We looked further into an unexpected, possible role of BadL in the generation of light-driven proton motive force (pmf) in R. palustris. For this purpose, we used qRT-PCR to measure transcript levels of *pucC* and *pufM.* PucC is a putative chlorophyll major facilitator superfamily exporter, while PufM is a photosynthetic reaction center subunit. Subsequent experiments were performed in the presence of succinate to allow cells to grow. When cells were grown photoheterotrophically with succinate and benzoate, a strain lacking BadL had significantly lower levels of *pucC* and *pufM* transcripts compared to the levels measured in the *badL*^+^ strain (Fig. 12A and C). To determine whether or not this result was related to BadM function, *pucC* and *pufM* transcripts were analyzed in a Δ*badL* Δ*badM* strain. In cells lacking *badL*, the presence or absence of *badM* did not change the levels of *pucC* or *pufM* transcripts (Fig. 12B and D). In addition to qRT-PCR, spectral analysis of LH1 and LH2 complexes were obtained in *badL*^+^, Δ*badL*, Δ*badM*, and Δ*badL* Δ*badM* strains. In agreement with the qRT-PCR results, the strain carrying a deletion of *badM* and *badL* did not display increased pigment synthesis relative to the *badL* strain (Fig. 13A), suggesting that the reduction in pigmentation in the Δ*badL* strain was unrelated to BadM function. To investigate whether acetamidobenzoates were involved in pigment synthesis or to determine whether BadL had different acetylation targets, pigment scans were obtained with *badL* cultures grown photoheterotrophically on benzoate supplemented with 2-, 3-, or 4-acetamidobenzoate. Absorbance readings related to LH1 and LH2 were partially restored to *badL*^+^ levels only when 4-acetamidobenzoate was added to the medium (Fig. 13B). This would suggest a new role for BadL in energy conservation or a secondary target for acetamidobenzoates that is directly or indirectly affecting pigment biosynthesis. These possibilities are under investigation.

## DISCUSSION

The degradation of lignin-derived aromatics (e.g., benzoate, hydroxybenzoate) under anoxic conditions in the presence of light has been extensively studied in R. palustris. The pathway is well defined, and so are the genes encoding the enzymes required to convert such compounds to acetyl-CoA (3). In addition, elegant system-wide studies of the regulation of expression of the benzoate acid degradation (*bad*) genes of this bacterium have been reported (4, 15). In spite of this wealth of information, the function of one gene, *badL,* remained enigmatic. In genome databases, *badL* is annotated as encoding a homologue of the yeast Gcn5-type histone *N-* acetyltransferase (GNAT) (PF00583).

Here, we present evidence in support of the physiological role of the BadL protein in the degradation of benzoate and reveal an additional, unexpected role for this protein in the generation of the light-driven proton motive force of this bacterium. Importantly, we show that the role of BadL in photosynthesis is independent of its role in benzoate catabolism.

### BadL is required for the expression of the genes encoding enzymes that dearomatize the benzene ring.

As shown by *in vivo* data (Fig. 2A and B), R. palustris requires BadL function to grow on benzoate as the source of carbon under photoheterotrophic conditions. Notably, BadL function is not required beyond the point of the pathway where intermediates have been dearomatized (Fig. 2C). On the basis of bioinformatic information (Fig. 5), we propose that the strategy of using an acetyltransferase to affect the function of the repressor is shared by Geobacter metallireducens*, Magnetospirillum magneticum*, and probably many other benzoate degraders. Prior to this work, it was known that AadR, a Crp-like regulator, activated transcription of the *badDEFGAB* operon and that AadR was itself regulated through an oxygen-sensing two-component system (16). We have shown that the repression of *badDEFGAB* is relieved through small-molecule acetylation and the binding of the acetylated product to BadM.

### BadL function generates acetamidobenzoates, which bind to BadM, leading to *badDEFGAB* operon derepression.

**FIG 14:**
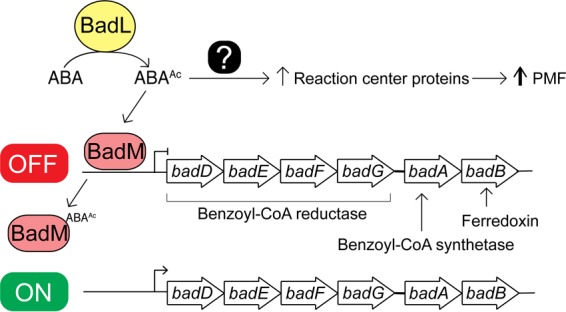
A model of the impact of acetamidobenzoates on benzoate degradation and photosynthesis. BadL acetylates aminobenzoates (ABAs) under photoheterotrophic growth. The resulting acetamidobenzoates (ABA^Ac^) either directly or indirectly increase the presence of reaction center proteins. Additionally, ABA^Ac^ binds to the BadM repressor, triggering expression of the *badDEFGAB* operon. The combined activities of benzoyl-CoA synthetase (BadA), benzoyl-CoA reductase (BadDEFG), and ferredoxin (BadB) activate and dearomatize the benzene ring. PMF, proton motive force.

The link between BadL function and the expression of genes encoding enzymes responsible for the activation of benzoate (EC 6.2.1.25) and its reduction to cyclohexa-1,5-diene-1-carbonyl-CoA (EC 1.3.7.8) suggested two possible functions for BadL. Either BadL modified and altered the function of BadA, BadB, BadDEFG, or BadM, or it acetylated a small molecule that would trigger the expression of the *badDEFGAB* genes. *In vivo* and *in vitro* evidence reported herein shows that the latter scenario is correct. BadL acetylates aminobenzoates (2-, 3-, or 4-ABA), yielding acetamidobenzoates (or 2-, 3-, or 4-ABA^Ac^) (Fig. 6 and 7), which bind to the BadM repressor, decreasing its affinity for its binding site upstream of the *badDEFGAB* operon (Fig. 8); this conclusion is supported by qRT-PCR data (Fig. 4A). While the concentration (10 mM) of ABA^Ac^ required in gel shift analyses was high, other studies characterizing Rrf2 regulators (37–39) utilized similar concentrations. An Rrf2 regulator, NsrR, senses nitric oxide (NO) and controls the expression of genes for NO metabolism. *In vitro* gel shift analyses utilized 10 to 20 mM concentrations of NO in order to completely abolish DNA binding (38). Additionally, studies that characterized regulators that bind aromatics as ligands used concentrations ranging from 0.1 to 10 mM (40, 41). One such study analyzed MobR, a repressor for the 3-hydroxybenzoate 4-hydroxylase gene in the soil bacterium, *Comamonas testosterone* (40). 3-Hydroxybenzoate acted as a ligand for MobR and was required at 10 mM concentrations in gel shift analyses (40). Furthermore, consistent with the idea that acetamidobenzoates act through BadM is the fact that in the absence of BadM, BadL function is irrelevant to benzoate degradation (Fig. 4B). A model of this hypothesis is shown in Fig. 14.

### Phenotypic analyses validate the assigned functionality of BadL as an aminobenzoate acetyltransferase.

Results of growth behavior analyses of mutant and wild-type strains offer strong support to the conclusion that, in R. palustris, acetamidobenzoates generated by BadL are required to relieve the BadM-dependent repression of the *badDEFGAB* operon. In other words, in the absence of BadL, the *badDEFGAB* operon is not expressed unless BadM is absent (Fig. 3A and 4). The fact that certain acetamidobenzoates present in the growth medium can bypass the need for BadL (Fig. 10) validates the role of BadL in benzoate degradation. Concerning the physiological relevance of each acetamidobenzoate derivative, we cannot say for certain whether a single acetamidobenzoate is the true ligand for BadM. All three acetamidobenzoates tested altered the affinity of BadM for the *badDEFGAB* promoter (Fig. 8 and 9), but addition of each acetamidobenzoate to the growth medium of Δ*badL* cultures led to different growth patterns. Notably, 4-acetamidobenzoate was not utilized as a carbon source (Fig. 10B), allowed the Δ*badL* strain to grow photoheterotrophically on benzoate (Fig. 10A), altered DNA binding of BadM (Fig. 9), and restored pigmentation of the Δ*badL* strain to wild-type levels (Fig. 13B). We would argue that in addition, 4-acetamidobenzoate may be used to allow for optimal growth of R. palustris under photosynthetic conditions.

### BadL activity is required for the expression of light-harvesting proteins.

The initial observation regarding the difference in pigmentation between Δ*badL* and *badL^+^* strains was intriguing because there was no reason to suspect a link between BadL function and energy conservation at the gene expression level. Since benzoate is catabolized by R. palustris only under photosynthetic conditions, it is reasonable that benzoate catabolism and photosynthesis would be linked in this organism. Clearly, the extraction of reducing equivalents from carbon sources requires that reduced electron carriers be oxidized so they can continually participate in carbon catabolism. In microorganisms that have electron transport systems, the oxidation of reduced electron carriers results in proton extrusion with the concomitant generation of a proton motive force. Data presented in this paper (Fig. 10 to 13) uncover an unexpected role for the BadL acetyltransferase in the regulation of expression of genes encoding proteins that are later assembled into light-harvesting complexes. To our knowledge, a connection between BadL function and *puc* and *puf* gene expression has not been reported. Previously, it has been shown that *puc* (RPA1547) expression is modulated through the Fix oxygen-sensing histidine kinase system (16). We find it intriguing that BadL would also be required for the transcription of *pucC*. We have shown that this is a BadM-independent process, and it has proved to be more complex for the scope of this paper. Additional experiments to find 4-ABA^Ac^ targets with regard to *puc* and *puf* expression must be performed. These experiments could include restoration of pigmentation in Δ*badL* strains by mutation or *in vitro* studies of interactions between 4-ABA^Ac^ and known pigment synthesis gene regulators.

Even though at this point we do not understand how BadL function is connected to energy generation, two points are worth discussing. First, we note that the observed decrease in light-harvesting 1 (LH1) and light-harvesting 2 (LH2) complexes in a Δ*badL* strain is not affected by the absence of the BadM repressor (Fig. 12 and 13A), and second, 4-acetamidobenzoate partially corrects the pigmentation phenotype (Fig. 13B), consistent with the need for the newly assigned BadL function as a source of acetamidobenzoates. This unexpected new role for acetamidobenzoates in the expression of genes encoding functions involved in reaction center biosynthesis is an exciting contribution to the field that warrants further investigation.

### Importance of BadL in Rhodopseudomonas palustris physiology.

From a physiological standpoint, the most intriguing question raised by this work is why does R. palustris rely on an *N-*acetyltransferase to control the transcription of genes required for photosynthetic growth on benzoate? One possible explanation is that aminobenzoates are very abundant in the environments occupied by R. palustris and that aminobenzoates can be readily deaminated to yield benzoate. Hence, high levels of aminobenzoates could have exerted a strong selective pressure for the evolution of a sensing mechanism that would activate the expression of genes encoding benzoate catabolic enzymes. At present, it appears as if the apparent lack of specificity of BadL for a given aminobenzoate may be an advantage, since maybe all aminobenzoates are used as a source of ammonia, yielding benzoate that can be used as carbon and energy. The connection of acetamidobenzoates to the expression of genes whose protein products are needed for the generation of a light-driven proton motive force is equally exciting, and further work in this area of R. palustris physiology will likely uncover new knowledge regarding the physiology of other bacteria that also appear to use *N*-acetyltransferases to regulate gene expression in response to diverse environmental stimuli.

## MATERIALS AND METHODS

### Chemicals, bacterial strains, culture media, and growth conditions.

All chemicals were purchased from Sigma-Aldrich with the following exceptions: [1-^14^C]acetyl-CoA (Moravek) (15 mCi mmol^−1^), 4-(2-hydroxyethyl)-1-piperazineethanesulfonic acid (HEPES) (GoldBio), isopropyl β-D-1-thiogalactopyranoside (IPTG) (GoldBio), ampicillin (GoldBio), 2-acetamidobenzoic acid (Alfa Aesar), 3- and 4-acetamidobenzoic acid (VWR). All strains and plasmids used in this study are listed in Tables S1 and S2 in the supplemental material. Escherichia coli strains DH5α (New England Biolabs) or C41(λDE3) (42) were grown on lysogeny broth (LB) (Difco) at 37°C. When used, antibiotics were added at the following concentrations: kanamycin (75 µg ml^−1^) and ampicillin (100 µg ml^−1^). All *Rhodopseudomonas* strains used in this study were derivatives of Rhodopseudomonas palustris CGA009 (43). For details on growth and pigment analysis of R. palustris, refer to Text S1 in the supplemental material.

10.1128/mBio.01895-18.3 TABLE S1 Bacterial strains and plasmids used in this study. Unless otherwise noted, all plasmids and strains were constructed in this study. Download Table S1, DOCX file, 0.01 MB. Copyright © 2018 VanDrisse and Escalante-Semerena.. 2018 VanDrisse and Escalante-Semerena This content is distributed under the terms of the Creative Commons Attribution 4.0 International license.

10.1128/mBio.01895-18.4 TABLE S2 Plasmids used in this study. Unless otherwise noted, all plasmids and strains were constructed in this study. Download Table S2, DOCX file, 0.02 MB. Copyright © 2018 VanDrisse and Escalante-Semerena.. 2018 VanDrisse and Escalante-Semerena This content is distributed under the terms of the Creative Commons Attribution 4.0 International license.

10.1128/mBio.01895-18.1 TEXT S1 Supplemental Materials and Methods. Download Text S1, PDF file, 0.1 MB. Copyright © 2018 VanDrisse and Escalante-Semerena.. 2018 VanDrisse and Escalante-Semerena This content is distributed under the terms of the Creative Commons Attribution 4.0 International license.

### Molecular techniques.

Primers were synthesized from Integrated DNA Technologies and are listed in Table S3. Genomic DNA was synthesized using ethanol precipitation (44). DNA manipulations were performed using standard techniques (45). DNA was amplified using Phusion High-Fidelity DNA polymerase (New England Biolabs) following the manufacturer’s protocol for amplification of high-GC DNA (GC buffer and 3% dimethyl sulfoxide [DMSO] [vol/vol]). PCR products were analyzed on agarose (1% [wt/vol]) gels developed at 100 V. PCR products were purified using the Wizard SV Gel and PCR Clean-Up System (Promega), and plasmids were purified using the Wizard Plus SV Miniprep kit (Promega). DNA sequencing was performed at the Georgia Genomics Facility (Athens, GA, USA). For details on plasmid construction for overexpression in E. coli or complementation in R. palustris, refer to Text S1.

10.1128/mBio.01895-18.5 TABLE S3 Primers used in this study. Download Table S3, DOCX file, 0.02 MB. Copyright © 2018 VanDrisse and Escalante-Semerena.. 2018 VanDrisse and Escalante-Semerena This content is distributed under the terms of the Creative Commons Attribution 4.0 International license.

### Purification of BadM and BadL proteins.

Plasmids p*Rp*BadM8, p*Rp*BadL1, p*Gm*BadL1, and p*Mm*BadL2 were transformed into E. coli C41(λDE3) *pka12*::*kan*^+^ (JE9314) cells. The resulting strains were grown overnight in 50 ml LB plus ampicillin. The cultures grown overnight were subcultured (1:100) into 2 liters of LB plus ampicillin and grown at 25°C to an optical density at 650 nm (OD_650_) of 0.5, after which transcription of genes was induced by the addition of IPTG (0.5 mM). Cells were harvested the next day by centrifugation at 6,000 × *g* for 15 min at 4°C in an Avanti J-2 XPI centrifuge equipped with rotor JLA-8.1000 (Beckman Coulter). Cell pellets were stored at −80°C until used. For further information on protein purifications and subsequent enzyme assays, refer to Text S1.

### DNA-binding assays.

Electrophoretic mobility shift assays (EMSAs) were performed using DNA probes containing a 6-carboxyfluorescein (6-FAM) label covalently attached to the 3′ ends of synthesized PCR products. Probes were generated using primers listed in Table S3 and included the intergenic region (212 bp) of *badC* and *badD* as described previously (15). Further details can be found in Text S1.

### RNA isolation.

Strains JE11529 (*badL^+^*/pBBR1-MCS2), JE13235 (Δ*badL*/pBBR1-MCS2), and JE13236 (Δ*badL*/p*Rp*BadL3) were each grown in quadruplicate in 5 ml of YP medium plus kanamycin for 4 days. Cells were diluted (1:20 [vol/vol]) into 5 ml of fresh PM plus succinate and grown photosynthetically until cells reached mid-exponential phase (OD_660_ of ∼0.5, 24 h later). Exponentially growing cells were back diluted into 15 ml of fresh PM plus succinate to an OD_660_ of 0.03 and grown photosynthetically at 30 °C until the cultures reached an OD of 0.1, at which point benzoate (3 mM) and NaHCO_3_ (10 mM) were added to the cultures with a sterile syringe and needle (15). Cells were grown for 24 h after the addition of benzoate, and all 15 ml was harvested by centrifugation at 4,000 × *g* for 10 min. Supernatants were decanted, and cells were immediately centrifuged at 4,000 × *g* for 1 min. Excess medium was aspirated off, and cells were flash frozen and stored at −80°C until used. RNA was isolated using the RNAsnap method (46). For RNAsnap protocol and cDNA synthesis procedure, refer to Text S1.
