# Supplementary figures and images for: Small-Molecule Acetylation Controls the Degradation of Benzoate and Photosynthesis in Rhodopseudomonas palustris
Source: mBio. 2018 Oct 16;9(5):e01895-18. doi: 10.1128/mBio.01895-18 (PMC6191541; doi:10.1128/mBio.01895-18)

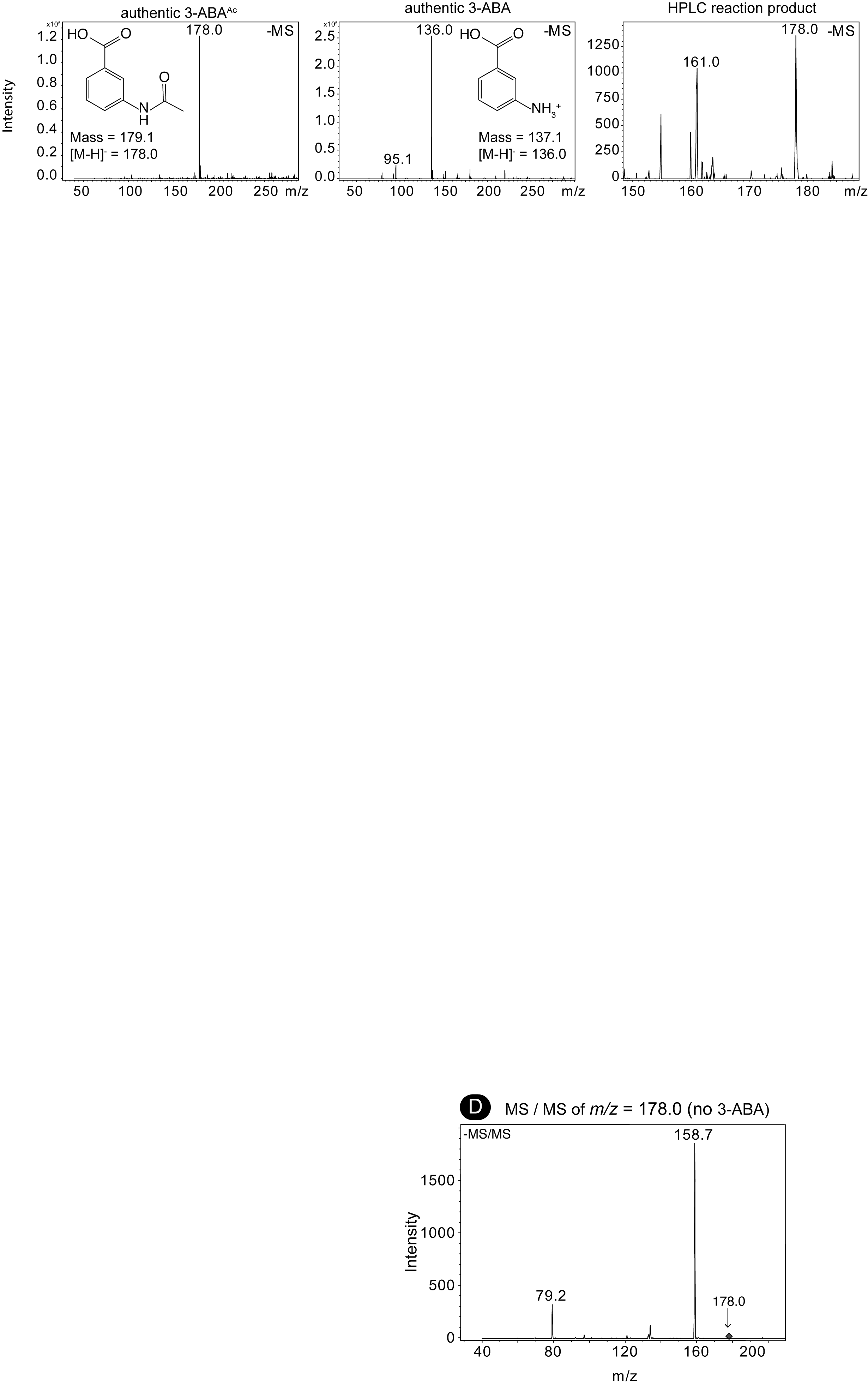

Supplement: FIG S1 [file mbo005184114sf1.tif]
