# Supplementary material for: Small-Molecule Acetylation Controls the Degradation of Benzoate and Photosynthesis in Rhodopseudomonas palustris
Source: mBio. 2018 Oct 16;9(5):e01895-18. doi: 10.1128/mBio.01895-18 (PMC6191541; doi:10.1128/mBio.01895-18)
Supplement: TABLE S1 [file mbo005184114st1.docx]

| **Table S1. Bacterial Strains and plasmids used in this study** | | |
| --- | --- | --- |
| **Strain** | **Relative Genotype** | **Source^1^** |
| ***E. coli strains*** | | |
| *E. coli* DH5α | Φ80d*lacZ*ΔM15 *recA*1 *endA*1 *gyrA*96 *thi*-1 *hsdR*17 (r_k_-, m_k_^+^) *supE*44 *relA*1 *deoR* Δ(*lacZYA-argF)* U169 *phoA* | NEB |
| *E. coli* C41 (λDE3) | *pka12*::*kan^+^ ompT hsdS* (r_B_m_B_) *gal* (λDE3*)* | Laboratory collection |
| ***R. palustris strains*** | | |
| JE11365 | *R. palustris* CGA009 (wild-type strain) | C. Harwood |
| Derivatives of JE11365 |  |  |
| JE11529 | *badL^+^* / pBBR1MCS-2 |  |
| JE19690 | *badL^+^* / pAadR3 |  |
| JE12597 | Δ*badL* |  |
| JE13235 | Δ*badL* / pBBR1MCS-2 |  |
| JE13236 | Δ*badL* / pBadL3 |  |
| JE19220 | Δ*badM* |  |
| JE19534 | Δ*badM* / pBBR1MCS-2 |  |
| JE19221 | Δ*badL* Δ*badM* |  |
| JE19535 | Δ*badL* Δ*badM* / pBBR1MCS-2 |  |
| JE19537 | Δ*badL* Δ*badM* / pBadM3 |  |
| JE19691 | Δ*badL* / pAadR3 |  |

^1^Unless otherwise noted, all plasmids and strains were constructed in this study
