# Supplementary material for: Small-Molecule Acetylation Controls the Degradation of Benzoate and Photosynthesis in Rhodopseudomonas palustris
Source: mBio. 2018 Oct 16;9(5):e01895-18. doi: 10.1128/mBio.01895-18 (PMC6191541; doi:10.1128/mBio.01895-18)
Supplement: TABLE S2 [file mbo005184114st2.docx]

| **­Table S2. Plasmids used in this study** | | |
| --- | --- | --- |
| **Plasmid** | **Genotype** | **Source^1^** |
| pTEV6 | *lacI^+^ malE^+^ bla^+^* | (11) |
| pTEV18 | *lacI^+^ bla^+^* | (4) |
| pTEV20 | *lacI^+^ bla^+^* | (4) |
| pBBR1MCS-2 | *kan^+^* | (12) |
| pK18mobsacB | *kan^+^ sacB^+^* | (7) |
| **Overexpression plasmids** | | |
| p*Rp*BadL1 | *R. palustris badL*^+^ cloned into pTEV6, *bla^+^* |  |
| pBadM8 | *R. palustris badM*^+^ cloned into pTEV20, *bla^+^* |  |
| p*Mm*BadL | *M. magneticum badL* ^+^ cloned into pTEV18, *bla*^+^ |  |
| p*Gm*BadL | *G. metallireducens badL* ^+^ cloned into pTEV18, *bla*^+^ |  |
| **Complementation plasmids** | | |
| pBadL3 | *R. palustris badL*^+^ cloned into pBBR1MCS-2, *kan^+^* |  |
| pBadM3 | *R. palustris badM*^+^ cloned into pBBR1MCS-2, *kan^+^* |  |
| pAadR3 | *R. palustris aadR*^+^ cloned into pBBR1MCS-2, *kan^+^* |  |
| ***R. palustris* deletion constructs** | | |
| pBadL2 | Upstream and downstream regions of *badL* cloned into pK18mobsacB |  |
| pBadM2 | Upstream and downstream regions of *badM* cloned into pK18mobsacB |  |
| pBadLM1 | Upstream and downstream regions of *badLbadM* cloned into pK18mobsacB |  |

^1^Unless otherwise noted, all plasmids and strains were constructed in this study
