# Supplementary material for: Small-Molecule Acetylation Controls the Degradation of Benzoate and Photosynthesis in Rhodopseudomonas palustris
Source: mBio. 2018 Oct 16;9(5):e01895-18. doi: 10.1128/mBio.01895-18 (PMC6191541; doi:10.1128/mBio.01895-18)
Supplement: TEXT S1 [file mbo005184114s1.pdf]

SUPPLEMENTAL MATERIAL

Small molecule acetylation controls the degradation of benzoate and photosynthesis in *Rhodopseudomonas palustris*

Chelsey M. VanDrisse and Jorge C. Escalante-Semerena[1]
Department of Microbiology, University of Georgia, Athens, GA 30602, USA.

Running title: acetylation links carbon utilization to photosynthesis

[1]Corresponding author: Jorge C. Escalante-Semerena, jcescala@uga.edu

SUPPLEMENTAL MATERIALS AND METHODS.

Rhodopseudomonas palustris *culture media and growth conditions.*

Rhodopseudomonas palustris was grown at 30 °C on YP rich medium (10 g yeast extract L^{-1} and 20 g peptone L^{-1}) or photosynthetic medium [PM (1)] supplemented with succinate (10 mM, pH 7 at 25°C) or benzoate (3 mM, pH 7 at 25°C) with $NaHCO_3$ (10 mM). When specified, kanamycin was added at 75 μg mL^{-1}. When added, ABAs and acetamidobenzoates stock solutions (100 mM) were dissolved in water and the pH was adjusted with NaOH until all material was dissolved. ABAs were added to growth medium at a final concentration of 5 mM. For growth analysis, cells were grown aerobically in 5 mL of YP medium for three days and supplemented with kanamycin when needed. Cultures were inoculated (1:30, v/v; stationary culture:fresh medium) in triplicate into 5 mL of PM in Balch tubes (2) supplemented with specified carbon sources and antibiotics, as listed in the figure legends. Tubes were stoppered and aluminum seals were crimped in place. The head space was degassed with O_2-free N_2 gas for 15 min. Cells were incubated without shaking at 30 °C in a light chamber equipped with eight light bulbs (60W, 120V, 840 lumens) with tubes 50 cm from light source. Cell density was monitored at 660 nm using a Manostat Spec 20D and all growth curves were repeated at least three times. *Geobacter metallireducens* GS-15 genomic DNA was a gift from Daniel Bond (University of Minnesota) and *Magnetospirillum magneticum* AMB-1 genomic DNA was a gift from Arash Komeili (UC Berkeley).

Plasmid construction for overexpression and complementation.

The following genes with their locus tags were PCR amplified as described above from purified gDNA: *R. palustris badM* (RPA0663, TX73_RS03425), *R. palustris badL* (RPA0664, TX73_RS03430), *G. metallireducens badL* (gmet_2096), and *M. magneticum badL* (amb3392, AMB_RS17160). Gene products (*badM, G.m. badL* and *M.m. badL*) and overexpression vectors (pTEV20 or pTEV18) were digested with restriction enzyme BspQI and cloned using published protocols (3, 4). *R. palustris badL* gene and overexpression vector (pTEV6) were digested with KpnI and HindIII restriction sites as described (5). Plasmids were transformed into *E. coli* DH5α and plated on LB agar supplemented with ampicillin. Positive clones were screened via colony PCR using T7 promoter forward and reverse primers. The resulting plasmids were: p*Rp*BadM8 (BadM fused to a His_6 recombinant tobacco etch virus (rTEV) prtotease-cleavable *C*-terminal tag), p*Rp*BadL1 (BadL fused to a His_6-MBP rTEV-cleavable *N*-terminal tag), p*Gm*BadL1, and p*Mm*BadL2 (*Gm or Mm*BadL fused to a His_6 rTEV-cleavable *N*-terminal tag). rTEV was overproduced and purified in house using described protocols (6).

R. palustris badL, badM, and *aadR* (RPA4234, TX73_RS21595) were amplified and purified as described above. The *badM* and *aadR* genes were cloned into HindIII and XbaI sites of

pBBR1MCS-2, resulting in plasmids p*Rp*BadM3 and p*Rp*AadR3. The *badL* gene was cloned into KpnI and HindIII sites of pBBR1MCS2, resulting in plasmid p*Rp*BadL3. Ligated plasmids were transformed into *E. coli* DH5α and plated on LB agar supplemented with kanamycin. Clones of interest were screened via colony PCR using M13 forward and reverse primers.

In-frame deletions of *R. palustris badL* and *badM* genes were constructed using described methods (7). Briefly, 1-kb regions upstream and downstream of targeted genes were amplified and fused using overlap extension PCR (8). The PCR products were digested with either EcoRI and HindIII (*badL*), EcoRI and BamHI (*badM*), or XbaI and HindIII (*badL badM*) and cloned into plasmid pK18*mobsacB* (7). Ligated plasmids were transformed into *E. coli* DH5α and plated on LB agar supplemented with kanamycin. Clones of interest were screened via colony PCR using M13 forward and reverse primers. Deletion construct plasmids were electroporated into exponentially growing *R. palustris* (washed twice with 10% glycerol, v/v) and plated on PM-succinate agar plates + kanamycin. Cells were grown under photosynthetic conditions in an Annoxomat Jar (Spiral Biotech) until single colonies appeared. Cells were streaked to isolation on PM-succinate agar plates + kanamycin and grown under light and anoxia. Multiple colonies were then streaked to isolation for counterselection on PM-succinate + sucrose (10%, v/v, added after autoclaving) and grown under light and anoxic conditions. Single colonies were screened for acquisition of the deletion and deletions were confirmed via DNA sequencing. For construction of Δ*badL* Δ*badM*, both genes were deleted simultaneously in a *badL*$^+$ *badM*$^+$ *R. palustris* strain. For complementation of genes, plasmids constructed as described above were electroporated into *R. palustris* and selected for transformation on PM-succinate + kanamycin agar plates.

***Rp*BadL, *Mm*BadL, *Gm*BadL, and BadM protein purification**

Cell pellets were thawed and resuspended in 30 mL of buffer A [HEPES (50 mM, pH 7.5 at 4°C), NaCl (0.5 M), imidazole (20 mM), and glycerol (20% v/v)] with lysozyme (1 mg mL^{-1}), DNase (1 μg mL^{-1}), and protease inhibitor phenylmethanesulfonyl fluoride (PMSF, 0.5 mM). Cells were lysed on ice with two rounds of sonication [1 min (2 s, 50% duty)] using a Q500 Sonicator (VWR) at 60% amplitude. Cell lysates were clarified via centrifugation at 40,000*g* for 30 min at 4°C in an Avanti J-25I centrifuge (Beckman Coulter) equipped with rotor JA-25.50. Supernatants were filtered through a 0.45 μm filter (Millipore) and samples were applied at 4°C to a pre-equilibrated 1-mL HisPur nickel-nitrilotriacetic acid (Ni-NTA) resin (Thermo Scientific). The column was washed with 10 column volumes (CV) of buffer A, 5 CV of buffer B [HEPES (50 mM, pH 7.5 at 4°C), NaCl (0.5 M), imidazole (80 mM), and glycerol (20% v/v)], and 5 CV of buffer C [HEPES (50 mM, pH 7.5 at 4°C), NaCl (0.5 M), imidazole (0.5 M), and glycerol (20% v/v)]. Proteins were separated by SDS-PAGE and concentrations of target protein containing fractions were quantified on a NanoDrop (Thermo Fischer) using the extinction coefficient and molecular weight of the protein (ExPASy, ProtParam).

Proteins were cleaved at 25°C for 3 h with a 1:50 mg:mg ratio of rTEV to target protein. After cleavage, proteins were dialyzed in buffer D [HEPES (50 mM, pH 7.5 at 4°C), NaCl (0.5 M), EDTA (1 mM), and glycerol (20% v/v)], and twice in buffer A. After dialysis, cleaved proteins were applied to a pre-equilibrated 1 mL HisPur Ni-NTA resin. Proteins that did not bind to the column were collected and dialyzed against three buffers with decreasing concentrations of NaCl (400 mM, 0.25 M, 150 mM), with a final buffer composition of [HEPES (50 mM, pH 7.5 at 4°C), NaCl (0.15 M), and glycerol (20% v/v)]. Protein concentrations were determined with a NanoDrop and were flash frozen in liquid N_2 and stored at -80°C until used.

Thin-layer chromatography (TLC)

Reaction mixtures included HEPES (50 mM, pH 7.5 at 25°C), TCEP (0.5 mM), [1-^{14}C]-Acetyl-CoA (20 μM), aromatic substrates (as listed in figures, 5 mM), and BadL protein (3 μg). Reaction mixtures were incubated at 37°C for 1 h and 5 μL of each reaction was spotted and dried onto a

pre-scored silica gel plate with aluminum backing (Whatman Ltd). Plates were developed for 2-3 h in a pre-equilibrated chamber with *n*-butanol, acetic acid, and water (3:1:1). Plates were dried, exposed to a phosphor screen, and imaged using a Typhoon Trio+ Variable Mode Imager (GE Healtcare) and analyzed with ImageQuant v5.2 software.

HPLC and MS/MS analyses

Reactions containing BadL (10 μM), acetyl-CoA (50 μM), aminobenzoates (100 μM), TCEP (0.5 mM), and sodium phosphate buffer (pH 8 at 25° C) were incubated for 2 h at 37°C. BadL was removed by passing reactions over an Amicon Ultra 0.5 mL 10kDa molecular cut off centrifugal filter (Millipore). The product of BadL aminobenzoate acetylation was resolved by RP-HPLC using a Shimadzu Prominence UFLC with a Kinetex 5μm C18 column (150 mm x 4.6 mm; Phenomenex). The column was equilibrated at a flow rate of 0.5 ml min^{-1} with 5 column volumes of elution buffer [50 % (v/v, in water) acetonitrile] and 5 column volumes of wash buffer [10 mM sodium phosphate in water (pH 8 at 25° C)]. Samples (100 μl) were injected and the column was developed with wash buffer for 5 min and then a gradient to elution buffer over 10 min. Compounds were detected and compared to standards at 254 nm using a computer-controlled Shimadzu Nexera X2 SPD-30A diode array detector. Data were analyzed using Prism v6 (GraphPad). Fractions were collected in 0.5 mL increments and fractions containing peaks corresponding to acetamidobenzoate were analyzed by MS and MS/MS (Protein and Mass Spectrometry Facility, UGA, Athens, GA, USA). Electrospray ionization (ESI)-MS was performed in acetonitrile and resolved on an Esquire 3000 Plus (Bruker) Ion Trap Mass Spectrometer at 0.3 ml h^{-1}. Pure standards (3-ABA and 3-ABA^{Ac}, 1 mM) were suspended in DMSO and run as described above.

DNA binding assays

Binding reaction mixtures (25 μL) contained 6-FAM double-stranded DNA probe (50 ng, 0.303 pmol), BadM buffer [Tris (20 mM, pH 7.5 at 25°C), KCl (50 mM), dithiothreitol (1 mM), and glycerol (8% v/v)], bovine serum albumin (BSA, 100 μg mL^{-1}), BadM (0 or 1.82 pmol), and acetamidobenzoates (10 mM). All acetamidobenzoates used (*i.e.,* 100 mM stock solutions of 2-, 3-, and 4-acetamidobenzoates were prepared in DMSO (100%), added to the reaction tube (to generate a master mix for three reactions), and immediately diluted with the appropriate amount of water followed by the addition of buffer, BSA, and protein. If DMSO stocks were added to water, precipitation occurred, hence order of addition was important. Reactions were incubated at 37°C for 10 mins and 5 μL of glycerol (50%, v/v) was added to the mixture. A non-denaturing gel with Tris-HCl (365 mM, pH 8.6) and polyacrylamide (7.5% w/v) (Criterion, BioRad) was pre-developed for 30 min at 110 V with 0.5x TBE (Tris base, boric acid, EDTA) buffer. Reactions (20 μL) were pipetted into the wells of the pre-developed gel and resolved at 110 V for 2-3 h. Gels were imaged using a Typhoon Trio+ variable mode imager (GE Healthcare) at wavelength 488 nm (blue setting) and analyzed with ImageQuant v5.2 software.

RNAsnap protocol for isolation of RNA for RT-qPCR

Cell pellets were resuspended in 150 μL of boil solution [EDTA (18 mM), sodium dodecyl sulfate (SDS, 0.025% v/v), RNA-grade formamide (95% v/v), 2-mercaptoethanol (1 % v/v)] in water and transferred to 1.7-mL Eppendorf tubes. Mixtures were incubated at 100°C for 7 min and immediately centrifuged at 16,000*g* for 5 min. A sample (100 μL) of supernatant was transferred to a fresh 2.0-mL Eppendorf tube containing 400 μL of RNase-free water and 50 μL of 3M sodium acetate, pH 5.2. Following RNA dilution, 1.65 mL of ice-cold ethanol (100%) was added to the reaction mixtures and tubes were incubated at -80°C for at least 1 h. After DNA/RNA precipitation, reaction mixtures were poured into 1.7-mL Eppendorf tubes and centrifuged at

16,000 x *g* for 1 h. Supernatants were decanted and pellets were washed with 300 μL of ice-cold ethanol (70% v/v). Supernatants were decanted, tubes were immediately centrifuged, excess supernatant was decanted and tubes were allowed to dry upside down on Kimwipes for 20 min. DNA/RNA pellets were resuspended in 100 μL of RNase-free water and centrifuged at 16,000 x *g* to remove non-soluble debris. Subsequent DNase I treatment was carried out with 90 μL of the DNA/RNA suspension and the Ambion Turbo DNA-free kit (Thermo Fischer Scientific) according to manufacturer's instructions for rigorous DNase treatment. After DNA cleavage, a final sodium acetate-ethanol precipitation was performed as described above. Dried RNA pellets were resuspended in 100 μL of RNase-free water and frozen at -80°C in 20-μL aliquots. A small sample for each RNA prep was sent to the Georgia Genomics Facility (Athens, GA, USA) for quality control analysis using the RNA 600 Nano kit of the Agilent 2100 bioanalyzer. Any RNA samples that had a RNA Integrity Number (RIN) of above 8.0 were used for subsequent qRT-PCR experiments.

cDNA synthesis and quantitative reverse-transcription PCR

Primers for qRT-PCR were designed using primer 3 software and were evaluated for specificity and melting curve. Total RNA (150 ng) from each sample was used with the iScript cDNA synthesis kit (BioRad) following the manufacturer's protocol. The final RNA concentration of the cDNA reaction mixture was 7.5 ng/μL and was used as a template for qRT-PCR. Master mixes were prepared in RNase-free water with FastSYBR green master mix (1X, Applied Biosystems) and gene-specific primers (1 μL of each primer from a 10 μM stock). qRT-PCR reactions (20 μL) were assembled with 15 ng of cDNA (2 μL of the 7.5 ng/μL cDNA) and 18 μL of the above-mentioned master mix into a MicroAmp™ Fast Optical 96-well reaction plate (Thermo Fischer Scientific). The real-time PCR was performed using a 7500 Fast real-time PCR system (Applied Biosystems). Cycle threshold (C_T) data were normalized to the *fixJ* (RPA4248) gene as described elsewhere (9). The normalized ΔC_T values were transformed using $2(e\text{-} \Delta C_T)/10^{-6}$ (10) and were reported as arbitrary expression units (EU). Mean EU values calculated from technical triplicates of biological triplicates were used to calculate the standard error of the mean in Prism6 software. Statistically significant differences between expression across strains were determined using a Welch's *t*-test with GraphPad Prism6 software. Figure legends report *p* values for each sample.

R. palustris *pigment analysis*

R. palustris strains were grown in triplicate for three days in YP medium aerobically. Cells were sub-cultured (1:30) into 10 mL of PM + benzoate (3 mM) and $NaHCO_3$ (10 mM). The headspace of Balch culture tubes was flushed with O_2-free N_2 gas, and cultures were grown photosynthetically as described above. At lag (OD_{660} ~0.20), log (OD_{660} ~0.6), and stationary phase (OD_{660} >1) of each replicate, 200 μL was removed from the Balch tube with a sterile needle and syringe. Culture samples (100 μL) were pipetted into a 96-well plate and scanned ($A_{600\text{-}1000}$) using a Spectramax UV-vis spectrophotometer. Replicates were plotted using GraphPad Prism6 software to determine mean and the standard error of the mean (SEM) for each strain.

REFERENCES

1. Kim M-K, Harwood CS. 1991. Regulation of benzoate-CoA ligase in *Rhodopseudomonas palustris*. FEMS Microbiol Lett 83:199-203.
2. Balch WE, Wolfe RS. 1976. New approach to the cultivation of methanogenic bacteria: 2-mercaptoethanesulfonic acid (HS-CoM)-dependent growth of *Methanobacterium ruminantium* in a pressurized atmosphere. Appl Environ Microbiol 32:781-791.
3. Galloway NR, Toutkoushian H, Nune M, Bose N, Momany C. 2013. Rapid cloning for protein crystallography using Type IIS restriction enzymes. Crystal Growth & Design 13:2833-2839.
4. VanDrisse CM, Escalante-Semerena JC. 2016. New high-cloning-efficiency vectors for complementation studies and recombinant protein overproduction in Escherichia coli and Salmonella enterica. Plasmid 86:1-6.
5. Elion EA, Marina P, Yu L. 2007. Constructing recombinant DNA molecules by PCR, p Unit 3.17.1-3.17.12. *In* Ausubel FM, R. Brent RE, Kingston DD, Moore JG, Seidman JA, Smith a, Struhl K (ed), Curr Protoc Mol Biol. Greene Publishing Associates & Wiley Interscience, New York, N.Y.
6. Blommel PG, Fox BG. 2007. A combined approach to improving large-scale production of tobacco etch virus protease. Protein Expr Purif 55:53-68.
7. Schafer A, Tauch A, Jager W, Kalinowski J, Thierbach G, Puhler A. 1994. Small mobilizable multi-purpose cloning vectors derived from the *Escherichia coli* plasmids pK18 and pK19: selection of defined deletions in the chromosome of *Corynebacterium glutamicum*. Gene 145:69-73.
8. Horton RM, Ho SN, Pullen JK, Hunt HD, Cai Z, Pease LR. 1993. Gene splicing by overlap extension. Methods Enzymol 217:270-279.
9. Egland PG, Gibson J, Harwood CS. 1995. Benzoate-coenzyme A ligase, encoded by *badA*, is one of 3 ligases able to catalyze benzoyl-coenzyme A formation during anaerobic growth of *Rhodopseudomonas palustris* on benzoate. J Bacteriol 177:6545-6551.
10. Livak KJ, Schmittgen TD. 2001. Analysis of relative gene expression data using real-time quantitative PCR and the 2(-Delta Delta C(T)) Method. Methods 25:402-408.
11. Rocco CJ, Dennison KL, Klenchin VA, Rayment I, Escalante-Semerena JC. 2008. Construction and use of new cloning vectors for the rapid isolation of recombinant proteins from *Escherichia coli*. Plasmid 59:231-237.
12. Kovach ME, Elzer PH, Hill DS, Robertson GT, Farris MA, Roop RM, 2nd, Peterson KM. 1995. Four new derivatives of the broad-host-range cloning vector pBBR1MCS, carrying different antibiotic-resistance cassettes. Gene 166:175-176.
