# Supplementary material for: Small-Molecule Acetylation Controls the Degradation of Benzoate and Photosynthesis in Rhodopseudomonas palustris
Source: mBio. 2018 Oct 16;9(5):e01895-18. doi: 10.1128/mBio.01895-18 (PMC6191541; doi:10.1128/mBio.01895-18)
Supplement: TABLE S3 [file mbo005184114st3.docx]

| **Table S3. Primers used in this study** | |
| --- | --- |
| **Primer Name** | **Primer Sequence^1^ 5’ 🡪 3’** |
| **Overexpression primers** | |
| 5’ *badL* pTEV6 KpnI | TTA**GGTACC**ATGAGTGGCGCAAGCAGCATC |
| 3’ *badL* pTEV6 HindIII | GGCG**AAGCTT**TCAGTCGAGATTCTTTTCCAT |
| 5’ *badM* pTEV20 | NN**GCTCTTC**NTACATGCCGATGCGGCTGCAGAAATC |
| 3’ *badM* pTEV20 | NN**GCTCTTC**NTTCCTAAGCCCGTCCGGCGGAGGAAT |
| 5’ *M.m. badL* pTEV18 | NN**GCTCTTC**NTTCATGGCAAATCCACAGGCTCGGCAAT |
| 3’ *M.m. badL* pTEV18 | NN**GCTCTTC**NTTATCAGAGATCCTTCTCGAACTGGATGAAG |
| 5’ *G.m. badL* pTEV18 | NN**GCTCTTC**NTTCATGAGCGATCAATTATCCATCCGTA |
| 3’ *G.m. badL* pTEV18 | NN**GCTCTTC**NTTATCAGTCGAGCGGTTTTTCCAACTCGAT |
| **Complementation primers** | |
| 5’ *badL* pBBR1MCS-2 | TTA**GGTACC**ATGAGTGGCGCAAGCAGCATC |
| 3’ *badL* pBBR1MCS-2 | GGCG**AAGCTT**TCAGTCGAGATTCTTTTCCAT |
| 5’ *badM* pBBR1MCS-2 HindIII | NNNNNN**AAGCTT**CGGTCGAGACCAACACGAAGATTG |
| 3’ *badM* pBBR1MCS-2 XbaI | NNNNNN**TCTAGA**CTAAGCCCGTCCGGCGGAGGAAT |
| 5’ *aadR* pBBR1MCS-2 HindIII | NNNNNN**AAGCTT**ATGCCGCATCTCGCTTATCCGA |
| 3’ *aadR* pBBR1MCS-2 XbaI | NNNNNN**TCTAGA**TCAGGCCGCGGCGAGCGCGT |
| ***R. palustris* deletion construct primers** | |
| 5’ upstream *badL* EcoRI | GTC**GAATTC**GATCGAGCACGACATGGAT |
| 3’ upstream *badL* | GACAACGCGATGAGTGGCGCAAGCTGATCGAAACGCCCGACACGCTCG |
| 5’ downstream *badL* | CGAGCGTGTCGGGCGTTTCGATCAGCTTGCGCCACTCATCGCGTTGTC |
| 3’ downstream *badL* HindIII | GAT**AAGCTT**GAACACGGCCTGACCAAG |
| 5’ upstream *badM* EcoRI | NNNNNN**GAATTC**CCGAACCTGTCGGTCGAAGAGAACC |
| 3’ upstream *badM* | CAATCTTCGTGTTGGTCTCGACCGCAAACCGCGCGAGCGTGTCGG |
| 5’ downstream *badM* | ACCTTAGCTGAACTTTCAGTCGGCAGCG |
| 3’ downstream *badM* BamHI | NNNNNN**GGATCC**TGCTGCACATCTTCCTGTCGAATTTGC |
| 5’ upstream *badLM* XbaI | NNNNNN**TCTAGA**TGCAGATCGACGAGCTCGACGTCA |
| 3’ upstream *badLM* | CGCGTTGTCCTCCCTCGTGGTGCCGCGTGATCCCGGCTCCTGATG |
| 5’ downstream *badLM* | CACCACGAGGGAGGACAACGCGACCTTAGCTGAACTTTCA GTC GGCAGCG |
| 3’ downstream *badLM* HindIII | NNNNNN**AAGCTT**TGCTGCACATCTTCCTGTCGAATTTGC |
| 5’ *badLM* del check | ACGAGATTCGGCGCAACGAAG |
| 3’ *badLM* del check | CAAGAGCGATCACGAGGTGCAG |
| **Electrophoretic Mobility Shift Assay Primers** | |
| 5’ *badCD* intergenic | AAGATCGCGATCGATATCTGAG |
| 3’ *badCD* intergenic | GTGATGCGGCCTGTCGAGC |
| **RT-qPCR primers** | |
| 5’ *badD* | ACCACGGCGGACATCATC |
| 3’ *badD* | GGTGAAACTGAGATCGGAGAACA |
| 5’ *fixJ* | GCGATGCGGGAGTCGAT |
| 3’ *fixJ* | TGTCTGCGCGGATTCGTA |
| 5’ *pucC* | CTTTACGCAGGTCCAGGTTG |
| 3’ *pucC* | GTTGAAGGTCGTCTGCGTTT |
| 5’ *pufM* | CGGCAATTGGTTACTGCTTC |
| 3’ *pufM* | CGTAGTTCGTTCGTGTCGTG |

^1^Bold nucleotides indicate restriction sites
